# The Biomechanics Behind Extreme Osteophagy in *Tyrannosaurus rex*

**DOI:** 10.1038/s41598-017-02161-w

**Published:** 2017-05-17

**Authors:** Paul M. Gignac, Gregory M. Erickson

**Affiliations:** 1 Department of Anatomy and Cell Biology, Oklahoma State University Center for Health Sciences, Tulsa, Oklahoma 74107-1898 USA; 2 Department of Biological Science, Florida State University, Tallahassee, Florida 32306-4295 USA

## Abstract

Most carnivorous mammals can pulverize skeletal elements by generating tooth pressures between occluding teeth that exceed cortical bone shear strength, thereby permitting access to marrow and phosphatic salts. Conversely, carnivorous reptiles have non-occluding dentitions that engender negligible bone damage during feeding. As a result, most reptilian predators can only consume bones in their entirety. Nevertheless, North American tyrannosaurids, including the giant (13 metres [m]) theropod dinosaur *Tyrannosaurus rex* stand out for habitually biting deeply into bones, pulverizing and digesting them. How this mammal-like capacity was possible, absent dental occlusion, is unknown. Here we analyzed *T*. *rex* feeding behaviour from trace evidence, estimated bite forces and tooth pressures, and studied tooth-bone contacts to provide the answer. We show that bone pulverization was made possible through a combination of: (1) prodigious bite forces (8,526–34,522 newtons [N]) and tooth pressures (718–2,974 megapascals [MPa]) promoting crack propagation in bones, (2) tooth form and dental arcade configurations that concentrated shear stresses, and (3) repetitive, localized biting. Collectively, these capacities and behaviors allowed *T*. *rex* to finely fragment bones and more fully exploit large dinosaur carcasses for sustenance relative to competing carnivores.

## Introduction

Most vertebrates cannot generate sufficient tooth pressures to gain access to marrow and phosphatic minerals trapped within the major bones of large animals. Carnivorous mammals (Carnivora) are the exception. Many, such as grey wolves (*Canis lupus*) and spotted hyenas (*Crocuta crocuta*), use their occluding incisors and cheek teeth to produce tooth pressures exceeding cortical bone shear strength to promote bone fragmentation^1–4^. (Note: bone is weakest in shear as opposed to compressional or tensional loading, and whole elements almost exclusively rupture via this mode^4, 5^). When necessary, mammals often employ repetitive, localized biting to finely comminute bones that are too large to swallow or crush. On the other hand, extant carnivorous reptiles (Sauria—including birds [Neornithes]) typically possess non-occluding teeth, or in the case of modern birds lack them entirely, and cannot generate sufficient stress distributions to fragment bones. Instead, they consume small carcasses in their entirety and large skeletal elements through dismemberment. Exceptions are: (1) Komodo dragons (*Varanus komodoensis*; Squamata) with ziphodont (recurved and serrated) teeth that occasionally leave shallow scores on skeletal elements^6, 7^ but do not crack them; (2) large, blunt-toothed crocodylians (e.g., American alligator—*Alligator mississippiensis*; Archosauria: Crocodylia), which puncture and occasionally crack bones when biting but do not finely fragment large sections of bones^8, 9^; and (3) some vultures (Accipitridae, Cathartidae) that drop bones on hard substrates to access marrow^10, 11^. Like their osteophagous carnivoran counterparts, these saurians have stomach acidity less than 1.5 pH^10, 12^, enabling chemical digestion of ingested bones.

**Figure 1:**
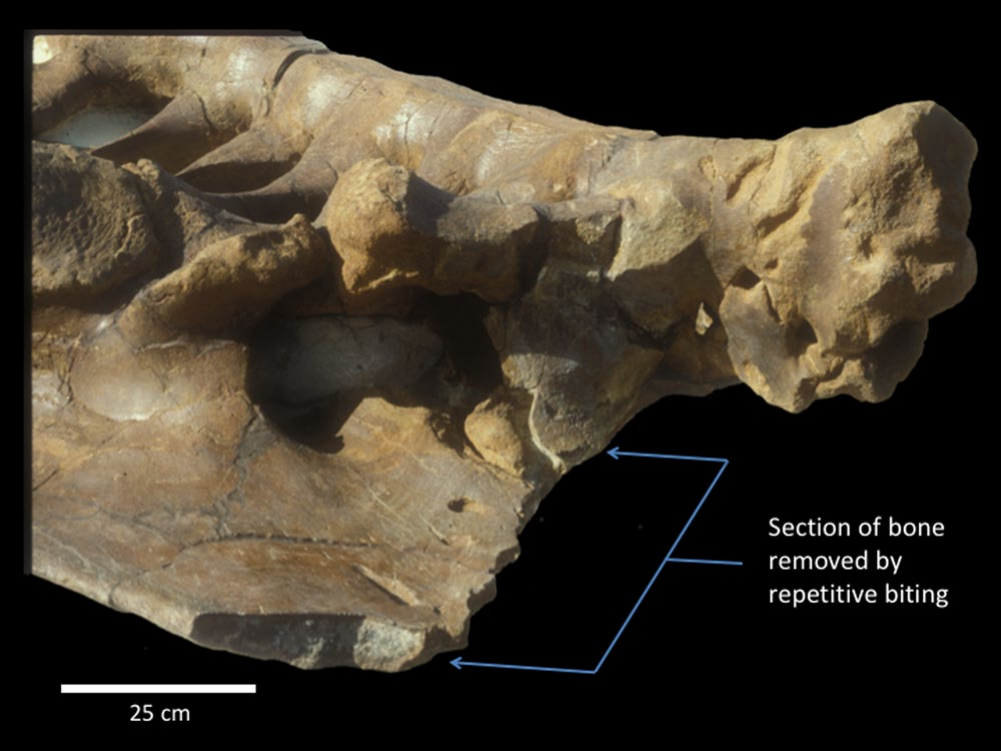
Left ilium of *Triceratops* sp. (MOR 799) in ventrolateral view with ~80 bite marks attributed to *Tyrannosaurus rex*. A large portion (~17%) of the iliac crest was removed (bracketed) by repetitive, localized biting.

Carnivorous dinosaurs (Archosauria: Theropoda), including most tyrannosaurids, also possessed ziphodont teeth and routinely made shallow scores and, occasionally, bone indentations during feeding^13–16^. However, extensive bite-mark evidence on herbivorous and conspecific dinosaur skeletons^15, 17–21^, heavily worn and broken teeth^16^, and bone-bearing coprolites^22, 23^ attributable to large (10–13 m) *Albertosaurus sarcophagus*, *Gorgosaurus libratus*, and *Tyrannosaurus rex* (Dinosauria: Tyrannosauridae), demonstrate that these North American taxa were exceptionally osteophagous, among theropods. All were equipped with large, stout lateral teeth (up to 18 centimetre [cm] crown length, 138 cubic centimetre [cc] volume in *T*. *rex*—the largest of any dinosaur^24^) that regularly scored, deeply punctured, and even sliced through bones^17^. Paradoxically, although these dinosaurs possessed non-occluding dentitions, they habitually finely fragmented bones—a capacity only known in mammals^25^. For example, an adult *Triceratops* sp. (Dinosauria: Ceratopsidae; Museum of the Rockies, Bozeman, Montana, USA [MOR] 799) bears ~80 *T*. *rex* bite marks^17^, revealing evidence of repetitive, localized biting and sequential bone removal around the left iliac crest (Fig. 1). In addition, tyrannosaurid coprolites^22, 23^ include hundreds of finely comminuted bone fragments that attest to this behavior. Bone fragments in a *T*. *rex* coprolite (e.g., Royal Saskatchewan Museum, Regina, Saskatchewan, CAN [SMNH] P2609.1) and from *Daspletosaurus* sp. stomach contents (Old Trail Museum, Choteau, Montana, USA [OTM] 200, 201) show rounding from acid dissolution, demonstrating that low pH acidity in tyrannosaurid stomachs allowed some sustenance to be liberated from the osseous ingesta^22, 26^.

Because trace evidence for extreme osteophagy is best documented in *T*. *rex*, we focus the present study on this taxon’s capacity to comminute bone. To do so requires a compound understanding of: (1) feeding behaviour; (2) tooth crown morphology; (3) forces applied through the teeth to create crack-initiating contact pressures (force/area^8^); and (4) consideration of how bones were contacted by individual and adjacent series of teeth. Of these factors, only the aforementioned trace evidence of feeding behaviour (see above) and adult bite forces for *T*. *rex* have been examined. However, with regard to bite-force values, disparate estimates have been reported. Specifically, Erickson and colleagues^25^ used an indentation simulation on cow pelves to estimate the force required to create a single *T*. *rex* bite mark during post-mortem feeding. This served to test the hypothesis that the taxon possessed structurally weak teeth. Although not assumed to be produced during maximal-force biting^25, 27^, a conservative value of 13,400 N (3,013 pounds [lb]) was obtained. At the time this was the highest experimentally derived bite force for any animal, suggesting the teeth were robust by neontological standards^25^. Because the contralateral teeth were similarly engaged during biting^25, 27^, the bite may have approached 26,800 N^27^ (6,025 lb). Meers^28^ subsequently used body-size scaling of bite-force values from a diversity of relatively small extant mammals and reptiles and derived an estimated maximal bite force of 253,123 N (56,907 lb) for an adult *T*. *rex* (MOR 555). Alternatively, Therrien *et al*.^29^ estimated maximal bite force by developing mandibular-bending profiles for one *A*. *mississippiensis* specimen and seven adult *T*. *rex* individuals (American Museum of Natural History, New York, New York, USA [AMNH] 5027; Black Hills Institute of Geological Research, Inc., Hill City, South Dakota, USA [BHI] 3033; Carnegie Museum of Natural History, Pittsburg, Pennsylvania, USA [CM] 9380; Field Museum of Natural History, Chicago, Illinois, USA [FMNH] PR 2081; Los Angeles County Museum, Los Angeles, California, USA [LACM] 23844; MOR 555, and the Royal Tyrrell Museum of Paleontology, Drumheller, Alberta, CAN [RTMP] 81.6.1). Profile contrasts were used to mathematically scale up an experimentally measured, adult *A*. *mississippiensis* bite-force value^30^ that was then doubled to account for the contralateral adductor musculature. The authors deduced a taxon-representative, maximal bite-force value of 300,984 N (67,667 lb). Finally, Bates and Falkingham^31^ estimated maximal bite forces for BHI 3033, using: (1) a computed tomography (CT) rendition of the cranium; (2) various musculoskeletal architectural configurations based on generalized functional groups for both squamate reptiles and crocodylians; (3) a mammalian appendicular muscle stress value; and (4) a biomechanical model predicting recoil on initial tooth impact that was assumed to occur in crocodylians. (Note: this is inconsistent with real-time, *in vivo* bite force readings^32^). The study arrived at maximum-force estimates ranging from 35,000–57,000 N (7,869–12,815 lb).

**Figure 2:**
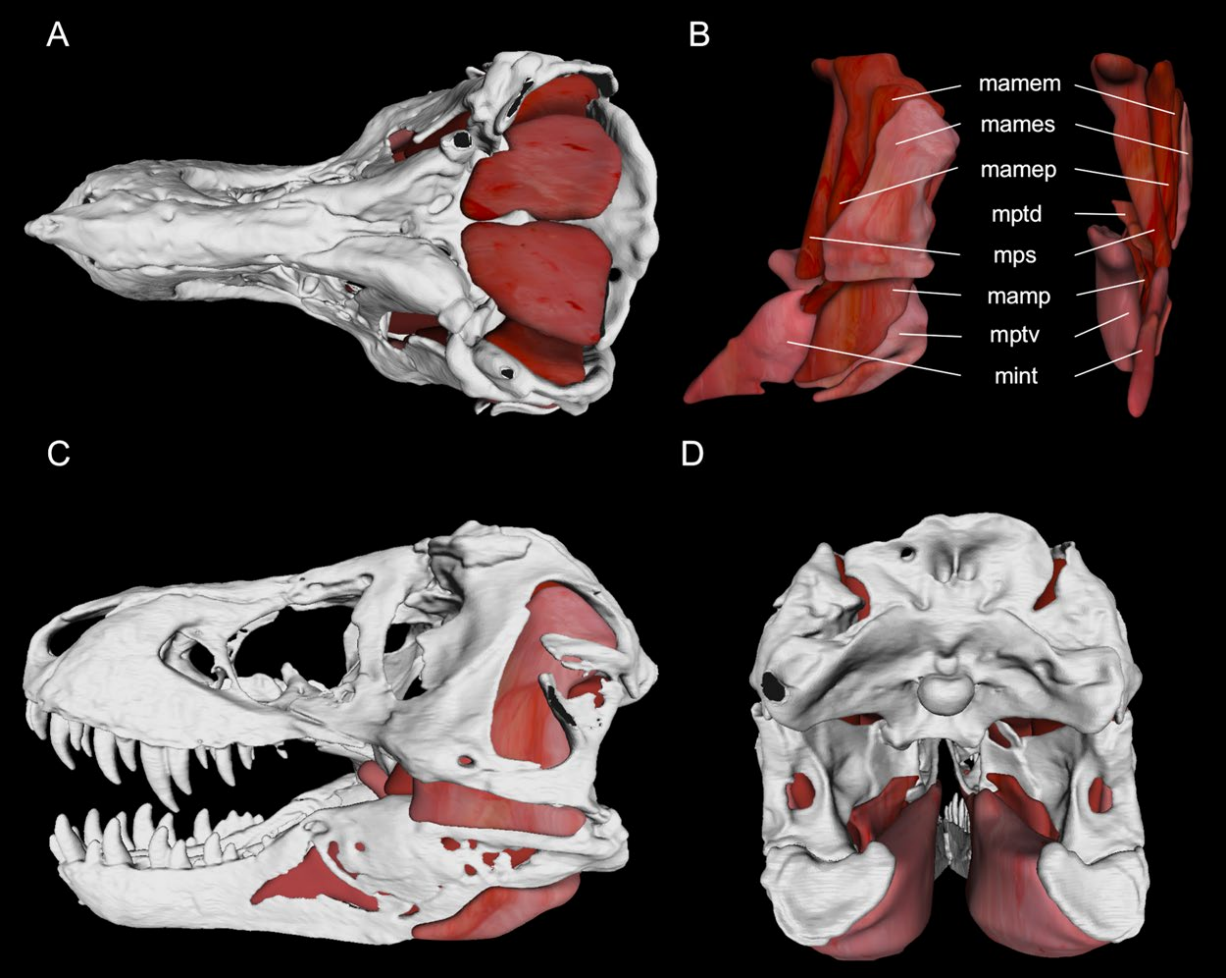
Jaw adductor muscle model for *Tyrannosaurus rex* (BHI 3033) in (**A**) dorsal, (**C**) left lateral, and (**D**) posterior views. Muscles in anatomical position are figured in (**B**) (lateral view is on left; anterior view is on right), textures and shades based on *Alligator mississippiensis* ^32^. Abbreviations: mamem, *Musculus adductor mandibulae externus medialis*; mames, *M*. *adductor mandibulae externus superficialis*; mamep, *M*. *adductor mandibulae externus profundus*; mptd, *M*. *pterygoideus dorsalis*; mps, *M*. *pseudotemporalis* complex; mamp, *M*. *adductor mandibulae posterior*; mptv, *M*. *pterygoideus ventralis;* mint, *M*. *intramandibularis*.

**Figure 3:**
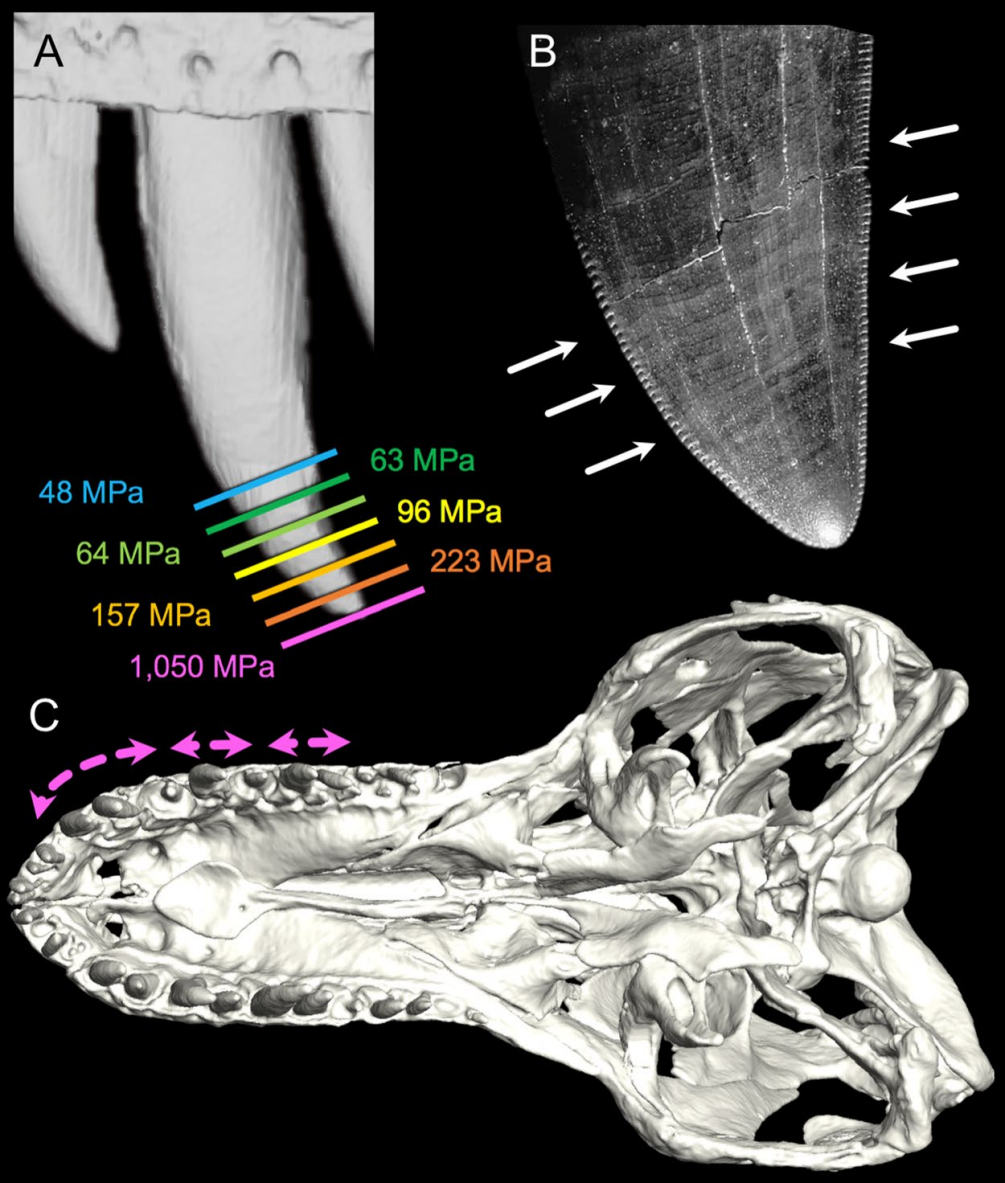
*Tyrannosaurus rex* dental functional morphology. (**A**) Exemplar tooth pressures along the distal 37 mm of the left M5 of BHI 3033 (warmer colours indicate higher pressures), illustrating bone-penetrating shear stresses (>65 MPa^4, 39^) for almost 25 mm of indentation depth. (**B**) Mesial and distal facing carinae (white arrows) helped direct pathways of bone fracture towards adjacent maxillary teeth (**C**) (ventral view of BHI 3033) that were also engaged during indentation, illustrating how the most procumbent maxillary tooth crowns collectively form a fracture arcade (pink arrows) due to pressures generated when biting. (Figure element in (**A**) derived from digital scan by Virtual Surfaces, Inc).

**Figure 4:**
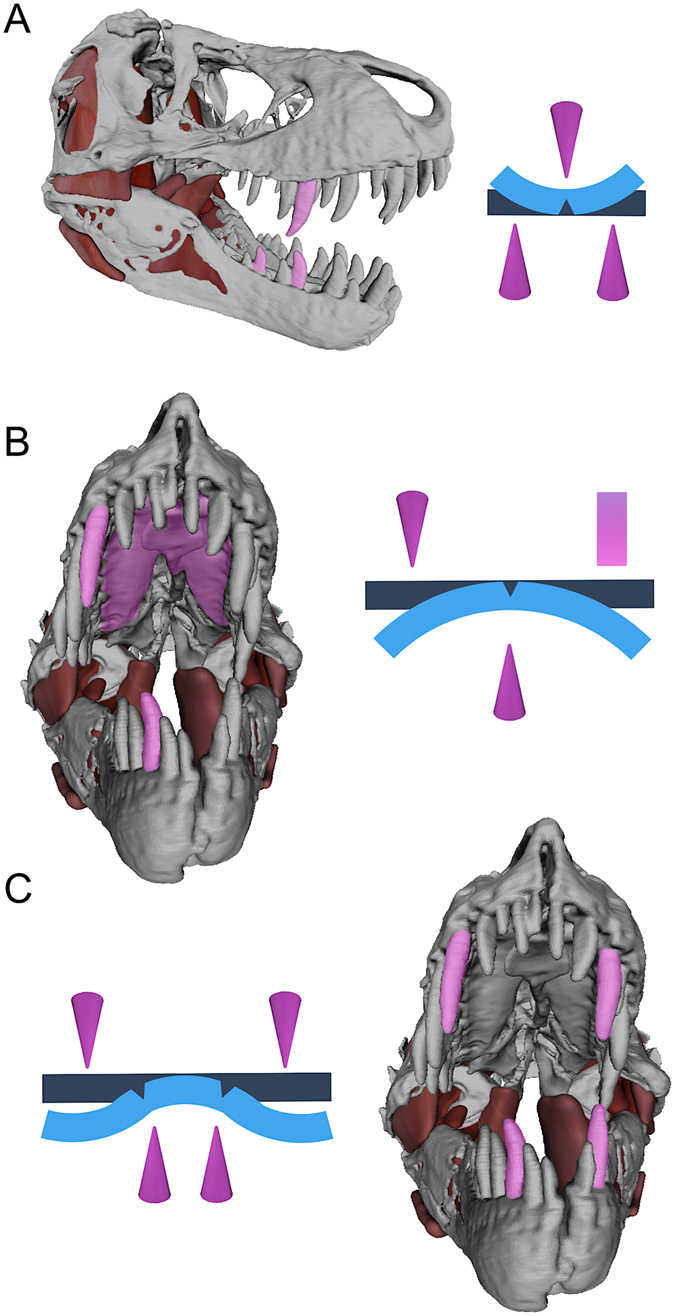
Jaw models of *Tyrannosaurus rex* paired with idealized beam diagrams, illustrating three- (**A**) (lateral view), (**B**) (anterior view) and four-point ((**C**), anterior view) loading configurations that allowed *T*. *rex* to promote failure stresses and fracture rigid structures (e.g., bone) without the aid of occluding dentitions. Teeth (cones) and the osseus palate, composed of the right and left maxillae and an anterior expansion of the vomer (rectangle), are shown as contact points in pink; original beam shapes are dark blue; and idealized plastic deformations (exaggerated) are light blue.

Because of the broad range of previous bite-force estimates for *T*. *rex* and an absence of data regarding applications of load and tooth pressures, we conducted a multifactorial examination of the biomechanics by which *T*. *rex* pulverized bone. Specifically we: (1) directly examined the crania and dentitions of specimens (n = 7) using articulated fossils, high-resolution museum-grade casts, and CT data spanning the entire known adult size range for the taxon; (2) characterized the contact areas of the prominent maxillary tooth crowns used to fracture bones during feeding^8, 33^; (3) reconstructed the three-dimensional (3-D), clade specific (Sauria: Archosauria) muscle architecture using Extant Phylogenetic Bracketing (i.e., inferring muscle configurations based on *T*. *rex* osteology and jaw adductors in Crocodylia—archosaurian sister clade to Dinosauria, and Neornithes—living theropod dinosaurs^34–36^) (Fig. 2); (4) determined muscle forces using an experimentally validated, extant archosaurian jaw adductor muscle model^35^; (5) size-scaled muscle forces and quantified specimen-specific lever mechanics of each jaw to estimate individual bite-force capacities; (6) deduced pressure generation as the teeth penetrated bones^33^ (Fig. 3A); and (7) considered the shear stress failure properties of bone to determine how dental and palatal contact configurations (Figs 3 and 4) facilitated skeletal element fragmentation in a manner consistent with *T*. *rex* bite marks and coprolitic evidence.

## Results

We found that adult *T*. *rex* skull lengths in our sample range from 111.5 to 136.5 cm (BHI 4100 and LACM 23844, respectively) (Table 1). Skull widths range from 59.2 to 90.2 cm (BHI 4100 and FMNH PR 2081, respectively) (Table 1). (Notably, FMNH PR 2081 has been reported as the largest specimen for the taxon^37^; however, we instead found that LACM 23844 has the longest and, marginally, the second widest skull). Based on the minimum reliable measurement of tooth contact areas (at 1 mm from the crown apex)^8^ and the deepest known *T*. *rex* tooth mark indentations (~37.5 mm)^15, 17^, we determined that maxillary tooth contact areas range from 6.3 to 565.1 mm^2^ (right M3 of MOR 980 and left M4 of RTMP 81.6.1, respectively) at minimum and maximum crown heights, respectively (Supplementary Table S2). Estimated bite forces range from up to 8,526 to 17,769 N (1,917 to 3,995 lb) mesially (right P1 of BHI 4100 and right and left P1 of FMNH PR 2081, respectively) and 18,014 N to 34,522 N (4,050 to 7,761 lb) distally (right M12 of BHI 4100 and right M12 of FMNH PR 2081, respectively) (Table 1). These are among the highest bite forces estimated for any animal (16,414 N [3,690 lb] was directly measured for a bob-tailed, 4.51 m Australian saltwater crocodile [*Crocodylus porosus*]^8, 38^). Apical tooth pressures (1 mm crown height) range from 718 to 2,974 MPa (104,137 to 431,342 pounds per square inch [psi]) (left M3 of BHI 4100 and right M5 of MOR 980, respectively) (Supplementary Table S2). The larger values are the highest tooth pressures ever estimated (2,473 MPa [358,678 psi] was deduced for a 2.99 m bob-tailed *C*. *porosus* ^8^). *Tyrannosaurus rex* tooth pressures exceeded the ultimate shear stress of cortical bone (65–71 MPa^4, 39^ [9,427–10,298 psi]) for at least 25 mm of crown height in nearly all maxillary teeth. One of the largest *T*. *rex* individuals (FMNH PR 2081) maintained such pressures up to (and presumably beyond) the 37 mm indentation maximum utilized in this study^15^.

**Table 1:** Adult^52^ *Tyrannosaurus rex* specimens, head lengths (HL), head widths (HW), and bite forces (BF, in newtons [N]) estimated in this study (see Methods and Supplementary Table S2 for measurement details and forces at specific tooth positions; see main text for museum abbreviations).

	HL (cm)	HW (cm)	BF range (N)
FMNH PR 2081	127.5	90.2	17,769–34,522
LACM 23844	136.5	89.0	16,352–31,284
MOR 980	128.2	81.5	14,201–30,487
MOR 008	116.2	79.6	13,736–28,101
BHI 3033	126.5	77.2	12,509–24,272
RTMP 81.6.1	117.2	70.5	12,197–21,799
BHI 4100	111.5	59.2	8,526–18,014

Analysis of dental-arcade configurations from *T*. *rex* skulls shows that its palatal and dental anatomy would have promoted: (1) fractures during biting that spanned between the mesial and distal carinae of adjacent teeth due to localized stress concentrations (Fig. 3); and (2) numerous three- and four-point loading configurations—classic means by which the tensional and shear weaknesses of beams (including bones) are exploited in mechanical and orthopaedic engineering with non-opposing loading points^4, 5, 40^. Three-point arrangements likely occurred: (1) between consecutive, large teeth along the dental arcade and the opposing tooth crown (Fig. 4A); and (2) between the lateral teeth and the anterior region of the bony palate, consisting of the right and left maxillae and an expanded portion of the fused vomers at the midline (see Fig. 4B), as can occur in other carnivores with reinforced palates such as crocodylians (P.M.G. and G.M.E., personal observations). Four-point loading likely occurred to bones spanning across both left and right upper and lower tooth rows (Fig. 4C).

## Discussion

Our findings, coupled with evidence of *T*. *rex* carcass utilization from bite marks, explain how this taxon along with other large North American tyrannosaurids comminuted bone in the absence of dental occlusion. The maximum adult *T*. *rex* bite forces (18,014–34,522 N; 4,050–7,761 lb) reported here for seven specimens spanning the adult size range for the taxon (see Table 1) are each moderately to considerably lower than previous estimates (35,000–300,984 N^28, 29, 31^; 7,869–67,667 lb). We suspect the differences stem primarily from previous models not implementing archosaurian-specific, jaw-closing musculature and force generation as well as not utilizing experimentally validated neontological models^35^. Nonetheless, the values we estimate are still prodigious. Adductor forces introduced tooth pressures substantially higher than the ultimate shear stress of cortical bone, even at great depth, allowing deep penetration of impacted bones. Tooth penetration served to drive open cracks (engendered first by localized fractures at tooth contact points), using broadly expanding tooth crowns^41^. Carinae accentuated these stresses and directed crack propagation towards adjacent teeth, resulting in high-pressure fracture arcades as cracks from the broadest and most procumbent teeth intersected during biting (Fig. 3). Together the dental and palatal anatomy also provided for three- and four-point loading configurations that facilitated localized and whole-element bone shear (Fig. 4). (Although not testable in our modelling, catastrophic explosion of some bones, particularly smaller elements or those with thin cortices, may have also occurred due to the introduction of strain energy densities exceeding the limits of bone^4^). Following fracture, repetitive and localized carnivoran-like biting (evidenced from bite marks; Fig. 1) served to accentuate fine-scale fragmentation, expose bone surfaces, and liberate marrow for rapid digestion by low pH stomach acids^22^.

The few osteophagous reptiles capable of driving cracks through bones, such as adult crocodylians^8, 33^ and tyrannosaurids, have force-resistant, thecodont dentitions. However, because of their characteristically offset dental rows, reptiles tend to generate a mechanical couple while biting (e.g., opposing but equal forces acting in parallel around a single axis; for an illustration see pages 19–20 in Cochran^5^), which can rotate isolated bones or those within carcasses and, potentially, load tooth crowns in unexpected ways. Such loads may induce reaction forces that can cause permanent structural failure^41–43^. Unexpected loads are counteracted by possessing semi-conical crowns with high, transverse-plane area moments of inertia. Such teeth are capable of sustaining comparable loads from any direction^30, 33, 44^, prolonging their functionality until replacement (e.g., over a year for large adult crocodylians^24, 45^ and ~777 days for *T*. *rex* ^24^) in these polyphodont taxa^24^. Taken together with the aforementioned prodigious bite forces, tooth pressures, localized biting, and absence of mammal-like, precise dental occlusion, our findings indicate that the extensive fragmentation of bone practiced by large tyrannosaurids was directly facilitated by their elongate, semi-conical, carinated, rooted, and polyphyodont dental arcades.

It is intriguing that the maximum tooth pressures shown here for *T*. *rex* overlap tightly with those reported for large adult crocodylians (e.g., *A*. *mississippiensis*, *C*. *porosus*) that are also capable of fracturing bone during feeding^8, 33^ (although not sequentially). Even though extant crocodylians are considerably smaller than adults of *T*. *rex*, both groups generate bone-failing pressures (e.g., crocodylian and *T*. *rex* tooth pressures at the distal crown of the most procumbent crushing teeth range from 309–2,473 MPa^8, 33^ [44,817–358,678 psi] and 718–2,974 MPa [104,137–431,342 psi] [Supplementary Table S2], respectively), using teeth with relatively thin enamel shells^16, 46^ (e.g., *A*. *mississippiensis* and *T*. *rex* mean ± standard error of enamel thicknesses sampled along the crown are 237 ± 6 and 223 ± 30 microns, respectively; GME unpublished data). In the case of crocodylians, the enamel shell is only slightly stronger than the tooth pressures that are typically endured during feeding (e.g., tooth safety factors—how mechanically overbuilt a structure is versus its function^4^—range from 1.0–1.4^8, 33^), and apical tip spalls^43^ are structurally similarly to those documented previously in *T*. *rex* ^16^. In the context of our integrative analysis, this functional convergence suggests that: (1) the performance capacities elucidated by this study are realistic; and (2) *T*. *rex* tooth crowns would be unlikely to sustain bite forces that are substantially greater^28, 29^ than those reported here. Notably, juvenile crocodylians with smaller and less robust dentitions are incapable of rupturing large bones^33^, which is also consistent with crown morphologies and bite marks from juvenile *T*. *rex* that similarly do not show evidence of bone removal. Instead these consist of only shallow punctures and scores^20^. Expansion of our protocol throughout ontogeny will help to elucidate at what size and age^25, 47^ this taxon’s capacity for bone fragmentation first occurred.

The collective results of this taxon’s biomechanical and physiological feeding capacities allowed these large-bodied theropods to uniquely exploit large bones from dinosaur carcasses—known to include giant horned-dinosaurs (e.g., *Triceratops* ^17, 18^), duck-billed hadrosaurids (e.g., *Edmontosaurus* ^15, 19^) and even other *T*. *rex* ^20^—that could not be consumed otherwise by contemporary carnivores. *Tyrannosaurus rex*, therefore, was able to derive sustenance from bones of prey^15^ and scavenged carcasses^27^, much like extant grey wolves^1–3^ and spotted hyenas^1, 3, 48^. Overall, our study shows how meaningful understanding of unusual behaviours and physical capacities not seen together in living animals can be determined through multifaceted, cross-disciplinary approaches. This research adds to a growing body of literature^49–51^ that illustrates how sophisticated feeding capacities—analogous to those of modern mammals and their immediate ancestors—were first achieved in Mesozoic archosaurs.

## Methods

### Specimen Examination

We examined fossil specimens, high-resolution museum-grade casts, computed tomography (CT) data, and professional photographs of the skulls, jaws, and dentitions of seven adult *T. rex* specimens (BHI 3033 [skull, cast, and CT], BHI 4100 [skull and cast], FMNH PR 2081 [cast], LACM 23844 [cast], MOR 008 [cast], MOR 980 [cast], RTMP 81.6.1 [skull and cast] at the AMNH and BHI). Adulthood in these individuals is based on corroborating information from craniofacial osteology^52^, overall size, and a mass-age growth curve^47^. We documented variation in head size using measures (linear distance to the nearest millimetre [mm; here and throughout our protocol]) of head width across the quadrates and head length from the anterior surface of the premaxillae to the posterior superior margin of the parietal bones. Individual variation in the lever mechanics of each skull was accounted for by measuring the linear distances between the quadrate-articular joint, and (1) the anteroposterior midpoint for osteological correlates of each jaw adductor muscle insertion along the lower jaw (i.e., “anatomical in-levers,” *sensu* ^35^); and (2) the midpoints for the first premaxillary alveolus (P1), the third, fourth, and fifth maxillary alveoli (M3, M4, M5, respectively), and the most distal maxillary alveolus (variably M11 or M12) on the left and right sides of the skulls (out-levers).

### Characterization of Tooth Contact Areas

Moulds were made of M3, M4, and M5 using fast-set silicon moulding putty (Knead-a-Mold^®^, Townsend Atelier, Chattanooga, TN, USA) on the right and left sides of all specimens for which teeth were fully erupted (the right M5 of BHI 4100 and left M4 of MOR 980 were not fully erupted and not used). These tooth crowns are the longest in the *T*. *rex* jaw and would, therefore, be the first to engage tissues in isolation during biting (and were determined to be responsible for the bite marks modelled previously^17, 25^). When present, M5 is typically the longest although either M3 or M4 may act to initiate tooth indentation when M5 is missing, broken, or beginning to erupt.

High-resolution epoxy replicas of the teeth were then made (Epoxyset #145–20005, Allied High Tech Products, Inc., Rancho Dominguez, California, USA). Crown heights and cross-sectional areas along each cast from the tooth apex towards the root of the crown were measured following^8, 33^. Cross-sectional measurements of conical and lenticular tooth crowns can serve as surrogates for realized tooth contact area (as demonstrated by Gignac and Erickson^33^), which sums the total indenter surface area that is in contact with indented tissues and perpendicular to the application of bite force through the long axis of the tooth. These measurements were ultimately used for estimating pressures generated along each tooth crown (see below)^8, 33^.

### 3-D Muscle Reconstruction

The actual, fully-articulated cranium, stereolithography files of individual skull bones (provided by BHI), and an articulated, high-resolution 14.45% scale replica rendered from the CT scans of *T*. *rex* specimen BHI 3033 were examined for making adductor muscle reconstructions. To further examine the adductor chambers and relationships of muscle attachment points, a micro-CT (μCT) scan of the articulated replica was undertaken at the Microscopy and Imaging Facility of the AMNH (2010 GE phoenix v|tome|x s240 high‐resolution microfocus CT system; General Electric, Fairfield, Connecticut, USA). A standard X‐ray scout image was obtained prior to scanning to confirm specimen orientation and define the scan volume. The scan was performed at 170 kilovolts [kV] and 145 micro-amps [μA], using a 0.1 mm copper filter, air as the background medium, and a tungsten target. The specimen was scanned at an isometric voxel size of 111.97 micrometres [μm] ( = 774.88 μm at life-sized dimensions), and slices were assembled on an HP z800 workstation (Hewlett‐Packard, Palo Alto, California, USA) running VG Studio Max (Volume Graphics GmbH, Heidelberg, Germany).

The specimen image stack was imported into Avizo Lite 9.0 (FEI Co., Hillsboro, Oregon, USA), where the skull and lower jaw were reconstructed separately. The lower jaw was abducted from the skull around the quadrate-articular joint to a standardized gape of 20° (measured from the anteroinferior margin of the premaxilla to the center of the quadrate-articular joint to the anterosuperior margin of the dentary). The skull and lower jaw were then resampled into one volume as a single material at an effective voxel size of 2.3513 mm (2,351.3 μm) to reduce file size and memory consumption for adductor muscle model generation as well as to scale the digital model to life-size dimensions. Although coarser, this abducted model retained bone-surface details necessary for use as an osteological scaffolding to reconstruct the jaw adductor musculature in three dimensions.

The eight adductor muscles (*Musculus adductor mandibulae externus medialis*, *M. adductor mandibulae externus profundus*, *M. adductor mandibulae externus superficialis*, *M. adductor mandibulae posterior*, *M. pseudotemporalis* complex, *M. intramandibularis*, *M. pterygoideus dorsalis*, and *M. pterygoideus ventralis*) that make up the archosaur jaw-closing system, based on extant Crocodylia and Aves^34, 35^, were rendered on both right and left sides (see Fig. 2). Based on comparisons to gross dissections^35^ and diffusible iodine-based contrast-enhanced CT (diceCT) specimens^53, 54^, the adductor muscle origins and insertions listed in Holliday^55^ (see Table 4 and in-text discussion) for the above jaw-closing muscles were regionalized in BHI 3033, and polygon volumes connecting those regions were rendered in Avizo Lite. Deviations in our model from the muscle attachments discussed by Holliday^55^ include the following: the two portions of *M. pseudotemporalis* were represented as a single muscle belly for model simplification that originated along the anteromedial, medial, and posterior portions of the upper temporal fenestra^35^, laterosphenoid^35, 55^ and epipterygoid^55^; *M. add. mand. ext. profundus* originated along the anterolateral, lateral, and posterolateral surfaces of the upper temporal fenestra; and *M. pterygoideus dorsalis* did not extend anteriorly past the orbits, as is the case for most birds^34^ but not for modern crocodylians^34, 35, 55^. In addition, the presence of a crocodylian-like, distinct *M. intramandibularis* in *T. rex* is unclear because it is hypothesized to have been fused to the *M. pseudotemporalis* musculature during the evolution of Avemetatarsalia (see Holliday^55^ for a detailed assessment). In this scenario the cartilaginous sesamoid (i.e., cartilago transiliens) that joined *M. pseudotemporalis* to *M. intramandibularis* was lost, resulting in a continuous muscle belly where there had once been two. In crocodylians the cartilago transiliens leaves a shallow fossa along the superiomedial surface of the mandible, adjacent to the pterygoid flange. Particularly well-preserved *T. rex* specimens such as FMNH PR 2081 (e.g., right mandible) may show faint evidence of such a depression (P.M.G., personal observation). Regardless, musculature attaching to the lower jaw and mandibular fossa in the position of the crocodylian *M. intramandibularis* and inferior avian *M. pseudotemporalis* ^34, 55^ was necessary for jaw adduction. Here we retained the crocodylian muscle topology based on trace evidence of this sesamoid cartilage. Lastly, *M. pterygoideus ventralis* was interpreted to wrap around the posteroinferior margin of the mandible and insert along the lateral surface of the lower jaw, inferior to the dorsolateral crest of the surangular (as depicted in Fig. 7C, left of Holliday^55^; also see Fig. 2C).

### Estimated Muscle Forces

Contractile forces for each adductor muscle were derived for BHI 3033 following a validated, free-body model analogue developed by Gignac and Erickson^35^ for *A. mississippiensis*. All muscles were assumed to have negligible pennation^55^, following the common fascicle configurations of both crocodylian^35^ and bird^34^ jaw adductor muscles. The exception to this is the uniquely pennate *M*. *pterygoideus ventralis* of crocodylians. The evolution of this muscle in eusuchians^56^ for high bite-force generation at the expense of adductor mandibulae and temporalis musculature (e.g., primary force generators in birds and other terrestrial amniotes)^34, 55^ promoted stealthy prey-capture behaviors at the water’s edge^35^. Modern crocodylians utilize this jaw adductor configuration for a substantially different feeding strategy than that inferred for *T*. *rex*. Without an *a priori* biological reason for assuming crocodylian-like pennation in *M*. *pterygoideus ventralis*, we modelled this muscle with a parallel fiber arrangement in *T*. *rex*.

To estimate physiological cross-sectional areas (PCSA) left- and right-side volumes for each muscle were (1) averaged, (2) divided by the density of archosaur skeletal muscle (1.056 gram [g]/cm^3^)^35^ to estimate mass of the contractile tissues, and (3) further divided by left-right average muscle lengths (see Gignac and Erickson^35^ for protocol quantifying 3-D muscle position) (Supplementary Table S1). As demonstrated by Gignac and Erickson^35^ muscle length can serve as a proxy for fascicle length in parallel-fibered muscles when statically modelled. Length measurements (Supplementary Table S1) were made as the 3-D linear distance between the centroids of each muscle’s origin and insertion. Each muscle was assigned an archosaur-specific muscle stress of 32.4 N/cm^2^ that was empirically determined from *A*. *mississippiensis* jaw adductor musculature^35^. (These values are not known for jaw muscles in Aves, which also lack teeth). Muscle forces were modelled assuming a tetanic contraction of 100% (Supplementary Table S1), which is broadly consistent with the maximum muscle recruitment values quantified by Cleuren *et al*.^57^ for *Caiman crocodilus* and comparable to Gignac and Erickson^35^ for *A*. *mississippiensis* through modelling of empirically derived bite-force values.

Jaw closure in *T*. *rex* is orthal with bite forces acting exclusively along the vertical (i.e., Y-oriented) axis of the teeth. Therefore, the dorsad-only component of each tetanic muscle force (Supplementary Table S1) was derived to estimate muscle moments at insertion points following the iterative Pythagorean Theorem approach of Gignac and Erickson^35^. This technique facilitates the ready transformation of tetanic muscle forces into muscle moments based on the anatomical in-lever lengths^35^ unique to each skull (Supplementary Table S2). The muscle-specific vertical components of contractile force for left and right-side jaw adductor muscle reconstructions in BHI 3033 were averaged (Supplementary Table S1) for use in estimating size-specific, Y-axis contractile muscle forces in the other adult *T*. *rex* specimens.

### Specimen Specific Bite-force Estimates

We used the tetanic muscle forces derived for BHI 3033 and bite-force relevant anatomical differences^35^ across our sample of skulls as the basis for estimating bite-force performance in the six-other adult *T*. *rex* specimens (Supplementary Table S2). Among living carnivorous archosaurs such as crocodylians, head width across the quadrates is a strong indicator of body size differences because it correlates strongly with body mass (Pearson’s product moment correlation = 0.99, n = 35)^56^. Therefore, we compared head width across the quadrates for our *T*. *rex* sample and linearly scaled the BHI 3033 vertical components of muscle force for each jaw adductor muscle to the values appropriate for BHI 4100, FMNH PR 2081, LACM 23844, MOR 008, MOR 980, and RTMP 81.6.1 based on these widths (Supplementary Table S2). Linear scaling (e.g., instead of a power curve) was used because the seven adult *T*. *rex* specimens occupy only a 34% range of total possible ontogenetic variation in head width, and linear versus power best-fit curves of muscle forces plotted against head width are not statistically distinguishable across such relatively small spans of body size in adult individuals of living animal models with low sample size (e.g., *A*. *mississippiensis* ^35^).

For each specimen, we then multiplied the scaled vertical component of muscle force into that muscle’s (right and left-side averaged) anatomical in-levers to produce muscle moments (Supplementary Table S2). The moments were summed, doubled (to account for the contralateral side jaw adductor musculature), and divided by each of the out-lever distances for P1, M3, M4, M5, and M11/12 tooth positions on the right and left sides to produce maximum bite-force estimations at the most mesial (P1) and distal (M11/12) tooth positions as well as for the most procumbent teeth (M3, M4, M5) (Supplementary Table S2).

### Tooth Pressures

Pressures along the tooth crowns of each individual were calculated by dividing the maximum estimated individual bite force at each tooth position by the tooth’s cross-sectional area measured at 1, 7, 13, 19, 25, 31 and 37 mm from the apex (Supplementary Table S2). These distances provided for the minimum cross-sectional area that could be measured reliably (1 mm), maximum indentation depth known for an adult *T*. *rex* bite mark (~37.5 mm)^15, 17^, and a series of regular intervals in between. Only tooth casts of pristine (undamaged) or nearly pristine (slightly worn) teeth were used in the analysis. These values were then compared to the ultimate shear stress of cortical bone^4, 39^. (Note: of the 31 tooth crowns we cast and measured, the following [see also Supplementary Table S2] were either blunted or showed fracture spalls at the crown apex and were not used in the apical tooth pressure analysis [BHI 3033 left M5 and right M4, BHI 4100 right M3 and M4, FMNH PR 2081 left M5, MOR 008 right M4, MOR 980 left M5 and right M4, and RTMP 81.6.1 left M4 and right M5]).

### Tooth and Palatal Contact Characterizations

The distribution and spacing of adult *T*. *rex* tooth crowns and palatal architecture were taken into consideration with regard to how they contributed to this animal’s ability to remove/comminute bones during biting. Specifically, we interpreted the loading of individual teeth (Fig. 3) as well as the collective damage invoked on bones by simultaneous indenting of both the upper and lower dentition in conjuction with potential palatal contacts (Figs 1, 3 and 4).

### Figures

Jaw adductor muscle figures were rendered by exporting polygon files from Avizo Lite into Meshlab version 1.3.3 (GNU General Public License version 2.0). Skull and muscle models were down-sampled in Meshlab using the Quadratic Edge Collapse Decimation tool. Colours were mapped to muscle polygons based on the phenotype of fresh adult *A. mississippiensis* jaw adductor musculature^32^.

## Electronic supplementary material

Datasets 1 and 2
